# A deep learning based ensemble approach for protein allergen classification

**DOI:** 10.7717/peerj-cs.1622

**Published:** 2023-10-12

**Authors:** Arun Kumar, Prashant Singh Rana

**Affiliations:** Computer Science and Engineering, Thapar Institute of Engineering and Technology, Patiala, Punjab, India

**Keywords:** Allergic reactions, Protein allergens, Bioinformatics, Machine learning, Deep learning, Ensemble learning

## Abstract

In recent years, the increased population has led to an increase in the demand for various industrially processed edibles and other consumable products. These industries regularly alter the proteins found in raw materials to generate more commercially viable end-products in order to keep up with consumer demand. These modifications result in a substance that may cause allergic reactions in consumers, thereby creating a protein allergen. The detection of such proteins in various substances is essential for the prevention, diagnosis and treatment of allergic conditions. Bioinformatics and computational methods can be used to analyze the information contained in amino-acid sequences to detect possible allergens. The article presents a deep learning based ensemble approach to identify protein allergens using Extra Tree, Deep Belief Network (DBN), and CatBoost models. The proposed ensemble model achieves higher detection accuracy by combining the prediction results of the three models using majority voting. The evaluation of the proposed model was carried out on the benchmark protein allergen dataset, and the performance analysis revealed that the proposed model outperforms the other state-of-the-art literature techniques with a protein allergen detection accuracy of 89.16%.

## Introduction

The prevalence of allergic reactions is a serious public health problem that may cause diseases like high fever, rhinitis, asthma, dermatitis *etc*., and affects a sizeable fraction of the world’s population (Pomés et al., 2018). Currently, the methods that can be used to cure allergies are not completely understood, and the only strategy that is known for preventing allergies is to abstain from substances that contain allergens (Sena-Torralba et al., 2020). However, the inaccurate detection of allergens may result in excessive dietary restrictions, which can then lead to nutritional issues (Shin et al., 2022). Hence it is clear that an efficient mechanism, which can amalgamate multiple parameters together for accurate allergen detection, is necessary for allergy control. The computational methods combined with bioinformatics have the ability to analyze multiple characteristics together for better allergen detection accuracy (Jeevanandam et al., 2022).

Protein allergens are a key contributor to the development of allergic reactions; hence, locating and describing these allergens is essential to the research and development of efficient diagnostic and therapeutic methods (Dimitrov et al., 2014a). Bioinformatics techniques may be used to conduct an analysis of protein allergen sequences in order to locate potentially allergenic areas because they contain carefully curated information on the sequences and structures of protein allergens (Yu et al., 2023). However, traditional techniques of allergen prediction take a lot of time and frequently depend on methods of experimentation that require a lot of manual resources (Zhou et al., 2019).

Machine learning and deep learning algorithms have only very recently emerged as potentially useful techniques for allergen prediction and classification (Westerhout et al., 2019; Bhardwaj et al., 2023). These algorithms are meant to analyse enormous volumes of data in order to recognise patterns that human analysts, depending on their level of experience, can miss. These intelligent algorithms have been utilised in a number of studies for the purpose of allergen prediction and categorization.

Individuals who are afflicted by allergies may benefit from a better diagnosis and treatment if researchers and clinicians include intelligent mechanisms in their work. So, in this article, we have described various essential factors/properties of protein sequences that can be used for the analysis and detection of different types of allergens.

The researchers have worked on various machine learning techniques for allergy detection and control. However, this article proposes a novel combination of ensemble learning, machine learning, and deep learning approaches for enhancing overall performance. Therefore, in this research, a deep learning based ensemble of three models: extra trees, DBN, and catboost has been proposed for the detection of protein allergens. The performance has been analyzed using different parameters like accuracy, F1-score, False Alarm Rate, Specificity, and Matthews correlation coefficient (MCC) *etc*.

The contribution of this article is summarised as:

- The article describes in detail the role and responsibilities of various entities related to allergy detection and control and also highlights the importance of protein allergen detection mechanisms.
- The features of the studied dataset, along with the relevance of each feature for allergen detection, have also been discussed in the article.
- A deep learning-based ensemble approach has been proposed, and its performance comparison with various state-of-the-art computational mechanisms for protein allergen detection has been presented.

The rest of the article is organized as: “Allergy Detection and Related Concepts” describes various concepts and entities that are essential for protein allergen classification; The recent state-of-the-art literature techniques pertaining to the field of allergy detection are discussed in “Related Work”; The analysis and working of the proposed mechanism is described in “Proposed Mechanism for Allergen Detection”; “Results and Discussion” presents the performance evaluation of various machine learning, deep learning and proposed mechanism for the detection of allergenic and non-allergenic protein sequences; Finally the article is concluded in “Conclusion”.

## Allergy detection and related concepts

This section describes in detail various entities and concepts related to allergies and mechanisms that can affect the detection of different types of protein allergen sequences.

### Preeminent allergy control organisations

The World Health Organisation (WHO) is a United Nations (UN) body charged with ensuring the health of people all over the globe. Its founding in 1948 was motivated by a desire to help people everywhere have happier and healthier lives in the years to come. By providing leadership and organizing efforts to prevent, diagnose, and cure illnesses, the WHO helps governments and other partners improve health outcomes. The WHO plays a pivotal role in the area of allergies by encouraging research and evidence-based policies to prevent and treat allergies and increasing awareness of the global effects of allergies (Bousquet et al., 1998).

For the greater good of the field, immunologists from all around the world have banded together to form the International Union of Immunological Societies (IUIS). The mission of IUIS is to develop the discipline of immunology *via* research, teaching, and advocacy, and to facilitate communication and collaboration among immunologists. IUIS has formed a subcommittee to standardize the nomenclature and categorization of allergens, known as the Allergen Nomenclature Subcommittee. The IUIS also keeps track of sequences, structures, and cross-reactivity data for allergens in their AllergenOnline database. When it comes to combating the problems caused by allergies, both the WHO and the IUIS play crucial responsibilities (Radauer et al., 2014). WHO has made supporting evidence-based policies to prevent and treat allergies a central part of its mission to improve international public health. The AllergenOnline database (a vetted repository of data on allergen sequences, structures, and cross-reactivity) and the IUIS’s work on allergen nomenclature are invaluable tools for allergy researchers (Goodman et al., 2016).

WHO and IUIS have been working together to combat the rising prevalence of allergic diseases. Increased measures to prevent and cure allergies have been advocated for by the WHO. Through its Global Initiative for Asthma, WHO has also created recommendations for the treatment of allergic reactions and asthma (Reddel et al., 2022). Most allergic reactions are brought on by protein allergens (Werfel et al., 2015). For accurate allergy diagnosis and therapy, it is necessary to first identify and characterize protein allergens.

### Allergen sequences

Allergens are compounds that, in those who are sensitive, can cause an allergic reaction. They come from many different places, such as pollens, moulds, foods, and animal dander. From minor itching and sneezing to severe anaphylaxis, allergens can induce a variety of symptoms that can be life-threatening (Meggs et al., 1996). As discussed previously in the article, one of the main causes of allergies is protein allergens. These allergens are proteins that can cause an immunological reaction in people who are prone to it. Protein allergens must be identified and characterized in order to create effective allergy diagnostic and management plans. The precise amino acid sequences that makeup protein allergens are known as protein allergen sequences. These allergen sequences are extremely varied, and even if they originate from the same source, various allergens may have distinct sequences. It is difficult to precisely detect and categorize allergies because of this heterogeneity (King et al., 1995).

A growing number of protein allergen sequences are being examined to find possible allergenic areas using bioinformatics methods. These technologies analyse the sequences using computer techniques to find potential immune response-inducing areas. Studying protein allergen sequences is crucial for understanding the mechanisms behind allergy reactions and creating effective diagnostic and treatment strategies. Using bioinformatics tools, standardising allergen naming and categorization, and applying machine learning and deep learning algorithms can help us learn more about protein allergens and their role in allergies.

### Machine learning and allergen identification

In protein allergen classification, machine learning is a powerful tool. Machine learning can help diagnose and manage allergies by using computational methods. The key role of machine learning in allergy control includes (Wang et al., 2021; Kavya et al., 2021):

- Machine learning systems can predict and detect allergenic proteins based on their sequence or structure. Training models using allergen data can predict allergenicity in newly found proteins and accelerate allergy diagnosis and treatment.
- Machine learning can extract amino acid composition, physicochemical features, and secondary or tertiary structural components from protein sequences and structures. These properties help to categorize allergic and non-allergenic proteins.
- When an allergic person reacts to structurally or functionally identical allergenic proteins, machine learning can predict cross-reactivity. Machine learning can assist in the design of hypoallergenic foods and allergy treatments by discovering cross-reactive protein families or motifs.
- Based on sequence or structural data, machine learning may identify allergenic protein sources (plant, animal, fungal). This can help discover allergies in new food or environmental samples.
- Machine learning can help personalize allergy diagnosis, treatment, and management by combining patient data like genetics and allergen exposure history.

Machine learning helps characterize and categorize protein allergens. Allergen identification, categorization, cross-reactivity prediction, and personalized medication improve allergy diagnosis, treatment, and management.

### Database

Data about allergens can be retrieved from a wide number of resources, such as the Allergen Nomenclature database maintained by the IUIS, the Structural Database of Allergenic Proteins (SDAP), and the AllergenOnline database. These databases include well-curated information on known allergens, including the sequences and structures of proteins (Dimitrov et al., 2014a).

The Protein Data Bank (PDB), the Swiss-Prot database, and the Structural Classification of Proteins (SCOP) database are some of the places where non-allergen data may be found (Sharma & Yadav, 2022). Other places where this data can be found include the Swiss-Prot database.

Data that does not pertain to allergens was included since it helps create a more well-rounded dataset for the purpose of training machine learning and deep learning algorithms. The inclusion of proteins that do not cause allergic reactions enables the algorithms to acquire the knowledge necessary to distinguish between allergenic and non-allergenic proteins based on their sequence features. Due to the fact that many proteins that are not allergenic may have sequence similarities to allergens, this is an extremely important factor for accurate allergenicity prediction.

Various properties are extracted from these allergen and non-allergen sequences as shown in Table 1. Peptide package available on the Comprehensive R Archive Network (CRAN) has been used for feature extraction (Osorio, Rondón-Villarreal & Torres, 2015). All the extracted features have been categorized as follows:

- Physicochemical properties: The term ‘physicochemical properties of protein sequences’ refers to the recognized physical and chemical properties of the amino acids that are used to form a protein. These characteristics have the potential to influence the structure, stability, and function of proteins, and they are essential for gaining a knowledge of the biological functions of proteins as well as creating medications and therapies that specifically target certain proteins (Osorio, Rondón-Villarreal & Torres, 2015).
- Molecular properties: The functions and behaviours of proteins at the molecular level are referred to as the molecular properties of protein sequences. Understanding protein function and interactions, as well as creating medications and therapies that target specific proteins, relies heavily on these characteristics (Osorio, Rondón-Villarreal & Torres, 2015).
- Structural descriptors: Protein structural features encompass the primary, secondary, tertiary, and Quaternary structure, as well as solvent accessibility, of the amino acid sequence. These characteristics are critical for understanding protein function and interaction with other molecules and for designing therapies and medicines with particular protein targets (Osorio, Rondón-Villarreal & Torres, 2015).

**Table 1:** Description of dataset features.

Feature descriptor	Category of descriptor	Method used for extraction of feature	Description
Amino acid indices	Physicochemical properties	aIndex (sequence)	The indices of amino acids are numerical values that stand for the physicochemical properties of certain amino acids. Some examples of these indices include hydrophobicity, polarity, and charge (Ikai, 1980).
Hydrophobicity scales	Physicochemical properties	Hydrophobicity (sequence, scale)	The hydrophobicity of amino acids is quantified using hydrophobicity scales. Optional prefix indicating whether hydrophobicity scale (KD, H, HW, MF, SH, or WS) is to be employed (R Graphical Manual and HR Documentation, 2014).
kideraFactors	Physicochemical properties	kideraFactors (sequence)	Amino acids are assigned numerical values called kidera factors that reflect their physicochemical traits. It returns the average of 10 kidera factors (Kidera et al., 1985).
Amino acid property scales	Physicochemical properties	stScales (sequence)	Amino acid property scales include ALIPHATIC, AROMATIC, BASIC, CYSTEINE, HYDROXYL, NEGATIVE, POSITIVE, SMALL, and TINY (Yang et al., 2010). ALIPHATIC rates alanine, valine, leucine, and isoleucine by aliphatic side chain size and shape. The AROMATIC scale ranks phenylalanine, tyrosine, and tryptophan based on their side chain aromatic rings. Because of their basic amino groups, lysine, arginine, and histidine have greater BASIC scores. All amino acids except cysteine are 0. Whether serine and threonine have hydroxyl groups (−OH) on their side chains affects HYDROXYL values. Aspartic acid and glutamic acid have negative ratings because their side chains include carboxyl groups. If lysine, arginine, or histidine possesses a positively charged amino group in its side chain, values are positive. SMALL scale values glycine, alanine, serine, threonine, and cysteine greater. Glycine and alanine, small amino acids, provide this scale’s eighth value.
VHSE scales	Physicochemical properties	vhseScales (sequence)	VHSE-scales (principal components score Vectors of Hydrophobic, Steric, and Electronic properties), are included in a total of 50 physicochemical variables of 20 coded amino acids and are derived from principal components analysis (PCA) on independent families of 18 hydrophobic properties, 17 steric properties, and 15 electronic properties, respectively (Mei et al., 2005).
Z scales	Physicochemical properties	zScales (sequence)	The physicochemical characteristics of the amino acids, such as NMR data and thin-layer chromatography (TLC) data, serve as the foundation for the Z-scales. Its value range from z1 to z4, reprsenting Lipophilicity, Steric, Electronic and other properties. It calculats mean of the Z-scales for each of the amino acids that make up the relevant peptide sequence (Sandberg et al., 1998).
Autocorrelation coefficients	Molecular descriptors	autoCorrelation (sequence, lag, property)	The autoCorrelation index is computed for a lag ‘d’ across a sequence of length ‘L’ using a descriptor ‘f’ (centred) (Cruciani et al., 2004).
Autocovariance coefficients	Molecular descriptors	autoCovariance (sequence, lag, property)	The autoCovariance index is computed for a lag ‘d’ across a sequence of length ‘L’ using a descriptor ‘f’ (centred) (Cruciani et al., 2004).
Cross-covariance coefficients	Molecular descriptors	crossCovariance (sequence, lag, property1, property2)	For a sequence of length ‘L’, the lagged crossCovariance index is computed for a lag ‘d’ using two descriptors, ‘f1’ and ‘f2’ (Cruciani et al., 2004).
Cruciani molecular descriptors	Molecular descriptors	crucianiProperties (sequence)	Using the scaled principal component scores that summarize a wide range of descriptors computed from the interaction of each amino acid residue with several chemical groups (or ‘probes’) like charged ions, methyl, hydroxyl groups, and so on, this function determines the Cruciani properties of an amino-acids sequence, which include: Polarity, Hydrophobicity, and Hydrogen Bonding (Cruciani et al., 2004).
WHIM molecular descriptors	Molecular descriptors	mswhimScores (sequence)	Twenty of the naturally occurring amino acids were used to generate 36 electrostatic potential attributes used to calculate TMS-WHIM scores (Zaliani & Gancia, 1999).
BLOSUM matrix scores	Sequence similarity	blosumIndices (sequence)	BLOSUM indices were created by employing an alignment matrix of the 20 natural amino acids and physicochemical attributes that had undergone VARIMAX investigations (Georgiev, 2009).
Boman index	Antimicrobial activity	Boman (sequence)	This function calculates Boman’s protein interaction index from a protein’s amino acid sequence. The index is the total of the solubility values for all residues in a sequence and can indicate a peptide’s capacity to attach to membranes or other proteins as receptors. This function predicts peptide-protein interactions (Boman, 2003).
Net charge at a given pH	Charge	Charge (sequence, pH = 7, pKscale)	Using the Henderson-Hasselbalch equation presented by D. S. Moore, this function determines the net charge of a protein sequence. There are nine different pKa scales that may be used to determine the net charge at a given pH, including Bjellqvist, Dawson, EMBOSS, Lehninger, Murray, Rodwell, Sillero, Solomon, and Stryer (Audain et al., 2016; Bjellqvist et al., 1993; Dawson et al., 2002; Gabernet et al., 2016; Lehninger, Nelson & Cox, 2005; Murray et al., 2003; Rodwell, 1982; Sillero & Maldonado, 2006; Solomons & Fryhle, 2008).
Isoelectric point	Charge	pI (sequence, pKscale)	When a certain molecule or surface has zero net electrical charge, the pH is said to be at its isoelectric point (pI). It is a factor that influences the solubility of the peptides at specific pH levels. A protein’s biological activity is typically lost when the pH of the solvent is the same as the protein’s pI pKscale—A string indicating which pK scale is to be used; valid values are ‘Bjellqvist’, ‘EMBOSS’, ‘Murray’, ‘Sillero’, ‘Solomon’, ‘Stryer’, ‘Lehninger’, ‘Dawson’, and ‘Rodwell’ (Audain et al., 2016; Bjellqvist et al., 1993; Dawson et al., 2002; Gabernet et al., 2016; Lehninger, Nelson & Cox, 2005; Murray et al., 2003; Rodwell, 1982; Sillero & Maldonado, 2006; Solomons & Fryhle, 2008).
Peptide length	Length	Lengthpep (seq)	The number of amino acids in a given protein sequence may be determined by using this function. Amino acids create long chains that are linked together by chemical compounds called peptide bonds to produce proteins (Kidera et al., 1985).
Mass shift due to modifications	Mass spectrometry	massShift (sequence, label, aaShift, monoisotopic)	Chemical changes or heavy isotope labelling change peptide mass. Function arguments: label—Label heavy isotopes. Accepts “none”, “silac_13c”, “silac_13c15n”, and “15n”. Replaces aaShift input. aaShift—Name the amino acid mass differential in Dalton vector. Names and values are amino acid one-letter codes and Dalton mass shifts. ‘Nterm’ and ‘Cterm’ define N- and C-terminal modifications. Monoisotopic: A logical value ‘TRUE’ or ‘FALSE’ indicating whether monoisotopic weights of amino acids should be utilised (Gasteiger et al., 2005).
Molecular weight	Mass spectrometry	mw (seq, monoisotopic, avgScale, label, aaShift)	It estimates protein sequence molecular weight. The scale on Compute pI/Mw tool calculates it as the total of each amino acid’s mass (Gasteiger et al., 2005). It calculates protein masses using predefined or custom stable isotope mass labels.
Mass-to-charge ratio	Mass spectrometry	mz (seq, charge, label, aaShift, cysteins)	Peptide mass-to-charge ratios (m/z) from mass spectrometry are determined using this function (Gasteiger et al., 2005).
Protein fingerprint	Structural features	protFP (seq)	Descriptors for the 20 naturally occurring amino acids were drawn from the AAindex database to form the basis of the ProtFP descriptor set (van Westen et al., 2013).

## Related work

Westerhout et al. (2019) talks about how hard it is to figure out how allergenic new proteins might be, and how we need to find new, safe sources of protein to make food in the future. The current rules for genetically edited proteins are based on a weight-of-evidence method, which looks at how similar the sequence is to known allergens, how resistant the protein is to being broken down by pepsin, and how it is linked to sugars. But other physical and biochemical features of proteins are not being looked at right now. In this study, the Random Forest method was used to make an *in silico* model that predicts the allergenic potential of a protein based on its physicochemical and biological features. The ProtParam tools and the PSIPred Protein Sequence Analysis programme were used to figure out 29 factors from the protein sequence.

Fernandez et al. (2021) talked about how important it is to make food allergy risk assessment systems that rank allergens based on how likely they are to cause an allergic reaction. This score will help the risk assessment process get more accurate knowledge about how allergens affect health. The study says that this method will improve on the present, too simple way of classifying proteins as allergens or not based on whether or not they are in an allergen database. Creating tailored biology tools based on better algorithms will make risk assessment methods more efficient and give the public more reliable information. The review comes to the conclusion that an international agreement on a more robust approach to allergen-sequence database curation is needed to improve the quality of allergenicity risk assessment of foods made with biotechnology and new foods, which is urgently needed in an age of climate change and the move towards more sustainable food systems.

The physiochemical and structural characteristics of allergen proteins originating from plants and animals were examined and compared in Behbahani, Rabiei & Mohabatkar (2020) using *in-silico* analysis and bioinformatics methods. The study attained an accuracy of 88.24% by analysing the attributes of the allergens and applying the PseAAC concept together with deep learning algorithms for categorization. In terms of their extinction coefficient and secondary structure, plant allergen proteins showed a more randomly coiled shape than animal allergen proteins, according to the investigation. The research shows the promise of bioinformatics-based methods for comparing allergens and elucidating their characteristics.

Omurca et al. (2019) designed an intelligent diagnostic assistant for predicting the type of an allergic disease across Turkey automatically by using well-known machine learning algorithms such as Decision Tree, Logistic Regression, Support Vector Machines (SVM), K nearest neighbour (kNN), and ensemble classifiers. This was accomplished by using Turkey as a case study. An allergic illnesses dataset, which originates from the Kocaeli University Research and Application Hospital, was utilised in the studies that were conducted. As a consequence of this, the highest accuracy rate of 77% was attained with majority vote when recognising 18 distinct allergy diagnoses.

In the context of sustainable food systems and goods produced from biotechnology, the scientific opinion in EFSA Panel on Genetically Modified Organisms (GMO) et al. (2022) discusses the urgent requirement for the advancement of allergenicity evaluation and protein safety. The ideas and recommendations that now underpin allergenicity risk assessment methodologies may not be in line with contemporary scientific developments. Significant information gaps still exist despite the European Food Safety Authority’s (EFSA) and EU-funded research programmes’ attempts to develop the area. The goals of this Scientific Opinion include identifying knowledge gaps in allergenicity prediction, pinpointing specific research requirements for enhancing allergenicity risk assessment, figuring out how recent discoveries and technological advancements can improve the current risk assessment methodologies, and prioritising research funding. The ‘weight-of-evidence’ method to evaluating allergenicity is still applicable, but the specific evidence needed will depend on the kind of biotech food under consideration. Improved gene and protein information standardisation, updated *in silico* tools, improved *in vitro* testing integration, and clearer guidance on the overall weight-of-evidence strategy for protein safety are among the major modernization areas. Future goods, such as those made using novel genomic methods and synthetic biology, will require the allergenicity risk assessment to change in order to handle their complexity. A road map that addresses important issues for risk assessors and management is required to clarify allergenicity safety objectives and risk assessment requirements.

The study in Singh et al. (2021) focuses on the most significant discoveries made in the field of food allergy research, which includes both computational biology and bioinformatics, as well as experimental investigations. It examines the present status of research prospects and future perspectives in the field of food allergy and offers an account of the tools and databases used for identifying and analysing food allergens. In addition, it provides an overview of the methods and databases used for identifying and analysing food allergens. The study highlights how important it is to identify the allergens that are present in various food sources in order to prevent unwanted effects and treat allergy illnesses that are caused by the intake of certain foods.

Nedyalkova et al. (2023) gives a detailed research endeavour that sought to create a proficient computational framework for forecasting the allergenic properties of proteins through the utilisation of diverse descriptors and machine learning algorithms. The research employed a dataset comprising proteins that are allergenic and non-allergenic, and conducted feature selection to determine the most pertinent descriptors. The performance of classifiers was assessed through cross-validation and diverse performance metrics. The findings indicate that KNN exhibited superior performance compared to alternative classifiers in distinguishing between allergenic and non-allergenic sequences. Conversely, SVM demonstrated better performance when utilising a limited number of descriptors. The study found that the chosen descriptors, which comprised of amino acid composition, evolutionary, and AAindex-based features, had a notable impact on the classification performance. The study showcased the potential of employing computational methodologies for the prediction of protein allergenicity. It also emphasised the significance of feature selection in enhancing the classification model’s efficacy.

The study in Yu et al. (2023) utilised Quantitative Structure Activity Relationship (QSAR) models to predict the binding ability of protein epitopes to IgE, the antibody responsible for allergic reactions. Four algorithms and selected chemical descriptors were used to establish models for predicting the binding capabilities of epitopes to IgE. The study validated the performance of the models through an Enzyme-Linked Immunosorbent Assay (ELISA). The results showed that the models were able to predict the allergic reactions of food protein epitopes effectively. According to the study, the amino acid sequence 116–130 of $\beta$-lactoglobulin ( $\beta$-LG) was identified as a new IgE-binding epitope through both *in vitro* experiments and the QSAR model.

Wang et al. (2021) proposed a mechanism for predicting the allergenicity of food proteins using deep learning models, specifically the pre-training BERT model, and novel ensemble learning models represented by LightGBM and XGBoost.

The study in Yang et al. (2020) concentrates on predicting human-virus protein-protein interactions (PPIs) using a random forest classifier based on doc2vec embeddings. This computational framework outperformed combinations of other widely-used machine learning algorithms and sequence encoding schemes, according to the study. The use of feature embedding for protein representation enabled the acquisition of additional context information from protein sequences, thereby enhancing prediction performance. The authors anticipate that their findings can be used to identify potential interactions between human and viral proteins and to direct experimental efforts to identify proteins implicated in human-virus interactions and their associated functional functions. The authors hypothesise that future advancements in deep learning architectures, protein structural information, and host PPI network topology can enhance the prediction of human-virus PPIs.

Sharma et al. (2021) used molecular descriptors and machine learning methods to create prediction models for chemical compound allergenicity. The PaDEL programme was used to compute the molecular descriptors for the 403 allergenic and 1,074 non-allergic substances that were taken from the IEDB database. On a dataset that included 2D, 3D, and FP descriptors, the models were trained and put to the test. The highest performance came from the hybrid descriptor-based Random Forest-based model, which on the validation dataset had an AUC of 0.93 and a maximum accuracy of 83.39%. It was discovered that several chemical fingerprints, such as GraphFP1014 and PubChemFP129, were more prevalent in allergens. The study also identified pharmaceuticals that are known to produce allergy symptoms and projected the probable allergenicity of FDA-approved medications. ChAlPred, a web server created for the project, enables the prediction and creation of compounds having allergenic qualities.

In comparison to previous computational techniques, AllerCatPro 2.0 (Nguyen et al., 2022) is a computational tool that has been designed to predict the allergenicity potential of proteins in food and personal care items. The similarity between input proteins and datasets of trustworthy proteins linked with allergenicity, which have been carefully selected from several sources, is assessed using both amino acid sequences and projected 3D structures. A total of 4,979 protein allergens, 162 mild allergenic proteins, and 165 autoimmune allergens are all included in these databases. Along with new features, AllerCatPro 2.0 offers more thorough results for possible cross-reactivity, protein information (UniProt/NCBI), functionality (Pfam, InterPro, SUPFAM), clinical relevance with regard to IgE prevalence, and allergen information pertaining to the allergen that is most similar. With an 84.7% sensitivity and 68.9% specificity on challenging benchmark datasets, the tool has been evaluated on numerous examples of profilins, autoimmune illnesses, low allergenic proteins, extremely big proteins, and nucleotide input sequences. It has demonstrated improved accuracy over earlier iterations.

Akbar et al. (2017) proposed an ensemble learning approach for the detection of anticancer peptides using genetic algorithm. The author also gives a detailed mathematical representation of the proposed ensemble technique. The authors have also extended the work in Akbar et al. (2021, 2023) for identifying antitubercular peptides and Neuropeptides by combining various machine learning techniques like Support Vector Machine, Random Forest, K-nearest neighbor *etc*., using ensemble learning.

MacMath, Chen & Khoury (2023) have reviewed the use of AI techniques for the detection, prevention, and cure of allergic reactions and other related tasks. The article also describes various challenges involved in the implementation AI techniques in such fields.

Zhang et al. (2023) presented a mechanism for screening of antihypertensive peptides using deep learning model. The effective results of the proposed deep learning model suggested that similar deep learning models can be used for related tasks involving peptides.

## Proposed mechanism for allergen detection

**Figure 1:**
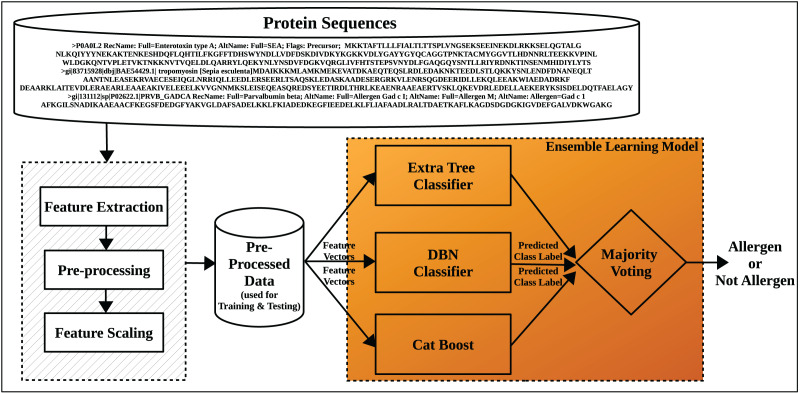
System model.

In this section we describe the details of proposed model for the detection of protein allergens. The literature suggests that by combining the outcome of different machine learning models together using ensemble learning techniques can enhance the overall accuracy of prediction. This work uses this concept and hence proposes an ensemble learning model to detect protein allergens in a given dataset. The system model for the proposed mechanism is shown in Fig. 1.

In the initial phase, we processed each protein sequence collected from different sources. The allergen protein sequences were accessed from AllergenOnline (Goodman et al., 2016), and non-allergen protein sequences were collected from National Center for Biotechnology Information (Wheeler et al., 2003), UniProt (Bairoch et al., 2005) and SWISS-PROT (Bairoch & Apweiler, 2000). These protein sequences were then processed for extracting features, that can be used to train different machine learning and deep learning models. In the Fig. 1 we have mentioned few protein sequences in the “Protein Sequence” block. The features were extracted using Peptide package available on CRAN. The physical, chemical and other properties, as described in Table 1, were extracted from each protein sequence.

The performance of any machine learning algorithm depends on the values contained in the dataset, and the prediction results are effective if all the feature values are numeric and lie in a particular range. In order to achieve this, we have replaced any missing value with the mean of the corresponding feature vector and encoded non-numeric values to numeric values using the labeled encoding technique.

The updated dataset was then passed to the feature scaling phase, where we used a standard scalar to transform all the values of feature vectors. If $x$ is the feature vector and $\bar x$ and $\sigma$ are the mean and standard deviation of the feature vector, then the transformed feature vector $\hat x$ is calculated using standard scalar as shown in Eq. 1.

(1) $$\hat x = {{(x - \bar x)} \over \sigma }$$

The completion of feature extraction, pre-processing and feature scaling results into a pre-processed dataset containing 128 features and 4,854 entries (among which 2,427 represent allergens and the rest represent non-allergens), which can then be used for training and testing the machine learning models.

We have selected three models for constructing the proposed ensemble learning model for detecting protein allergens. The selection was based on a thorough performance analysis of different machine learning and deep learning models on the dataset.

The proposed ensemble learning model contains a combination of Extra Tree, Deep Belief Network (DBN) and CatBoost models, the details of which are given as:

- **Extra Tree:** Decision tree algorithms are the basis of the Extra Trees (Extremely Randomised Trees) classifier, another ensemble learning approach. Training numerous decision trees and aggregating their predictions results in a more accurate and robust categorization. In comparison to the standard Random Forest algorithm, the Extra Trees classifier introduces additional randomness, leading to more diverse trees and improved generalisation performance (Polikar, 2006).
  The Extra Trees classifier generates a collection of decision trees, each of which is constructed using a different subset of the training data and a different set of characteristics at each node. Due to the algorithm’s lack of optimisation of the splitting criterion, it is both quicker and more random than conventional decision tree approaches like Random Forest, from which it borrows its name. By adding some randomness to the tree-building process, we may generate a more varied set of trees, which helps lower the model’s variance and boosts its generalisation performance. Rather of relying on just one tree’s predictions, the classifier aggregates the results from all of the trees in the ensemble, typically by majority vote, to provide more reliable and accurate classifications.
- **DBN:** To quickly and effectively create hierarchical representations of data, a DBN blends unsupervised and supervised learning approaches. DBNs are constructed from a number of layered Restricted Boltzmann Machines (RBMs). They are taught greedily, layer after layer, using unsupervised pre-training to set their starting weights and then supervised learning to fine-tune them. DBNs have been successful in a number of contexts, including those involving image and speech recognition, NLP, and dimensionality reduction (Lopes et al., 2015).
- **CatBoost:** A gradient boosting technique designed specifically for categorical features is called CatBoost. In addition to providing effective management of big datasets containing categorical variables, it is optimised for high-performance prediction (Bentéjac, Csörgő & Martínez-Muñoz, 2021).
  A gradient boosting technique designed specifically for categorical features is called CatBoost. In addition to providing effective management of big datasets containing categorical variables, it is optimised for high-performance prediction. To lessen target leakage and overfitting, it uses ordered boosting, which rearranges the training examples using a random permutation and then calculates target statistics for each category using the instances that came before them in the permutation.

As discussed earlier in this section, we have selected three models based on the performance analysis of various machine learning and deep learning models, to create an ensemble model. The other machine learning models used in the study include: Logistic Regression (with maximum iterations of 300 on L2 regularization and Limited-memory BFGS optimizer), k-Nearest Neighbour (with $k = 10$), Support Vector Machines (with three kernel variants: Linear, Polynomial of degree 3 and Radial Basis Function), Random Forest (with size of base estimator as 100 and criterion for node splitting as Gini index), Ada Boost, Gradient boosting and XG Boost (Zhou, 2021).

**Figure 2:**
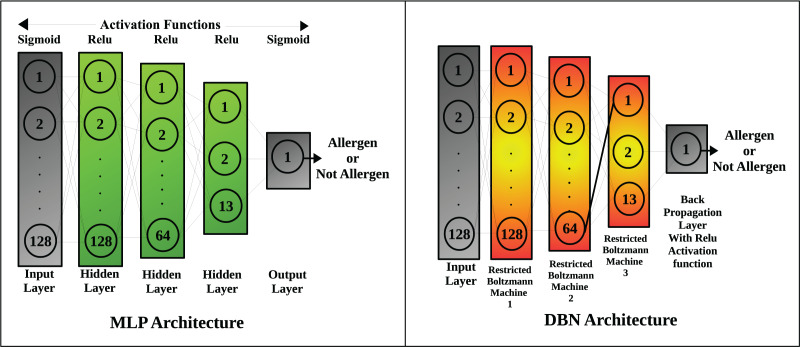
Architecture of neural networks used in the study.

The deep learning models include: Multi-layer perceptron and DBN, the architecture of which is shown in Fig. 2. The number of neurons in the input layer of each deep learning model equals the number of available features. The first hidden layer has the same number of neurons as that of the input layer, but the second hidden layer has 50% of neurons in the first hidden layer (*i.e*., 64 neurons). The third hidden layer has 13 neurons that are responsible for pattern recognition for major feature descriptors which include hydrophobicity, kideraFactors, amino acid property scales, VHSE scales, Z scales, Cruciani molecular descriptors, WHIM molecular descriptors, BLOSUM matrix scores, Boman index, charge, molecular weight, peptide length and protein fingerprint.

Each of the models in the proposed ensemble learning approach receives feature values concurrently and predicts the class label (allergen or not). The prediction results of the base models are then combined together using majority voting, the output of which gives the final predicted class label for the input values. In majority voting, the class label that receives the most votes becomes the ensemble’s prediction for that instance. We have used majority voting because it lowers the likelihood of overfitting, boosts model stability, and increases prediction accuracy (Polikar, 2012).

## Results and discussion

The performance analysis of the proposed model and other machine learning and deep learning models on the dataset was implemented using Python 3.10 with scikit-learn, PyTorch and other related packages on 1.6 GHz Intel Core i5 with 12 GB of RAM. The dataset was divided into training and testing set by random selection of feature values. The training and testing datasets contained 3,397 and 1,457 respectively, randomly selected entries.

### Performance parameters

In order to evaluate the performance of each learning model, we have trained each model on the training dataset and tested it on the testing dataset. The test results were calculated using confusion matrix (as shown in Table 2).

**Table 2:** Confusion matrix.

Actual value	Predicted value	
	Allergen	Non-allergen
**Allergen**	True positive (TP)	False negative (FN)
**Non-allergen**	False positive (FP)	True negative (TN)

Once the confusion matrix for each model on the testing dataset was evaluated, then the below-mentioned parameters were calculated to further analyze the performance:

(2) $$Accuracy = {{TP + TN} \over {TP + FN + FP + TN}}$$

(3) $$F1 - score = {{2*TP} \over {2*TP + FP + FN}}$$

(4) $$FalseAlarmRate = {{FP} \over {TN + FP}}$$

(5) $$Specificity = {{TN} \over {TN + FP}}$$

(6) $$MCC = {{(TP*TN) - (FP*FN)} \over {\sqrt {(TP + FN)*(TP + FP)*(TN + FP)*(TN + FN)} }}$$

Apart from the above mentioned parameters, we have also plotted Receiver Operating Characteristic (ROC) curve and Precision-Recall (PR) curve, and calculated Area Under the Curves (AUC). The training and testing time was also noted during the analysis.

### Result analysis

**Figure 3:**
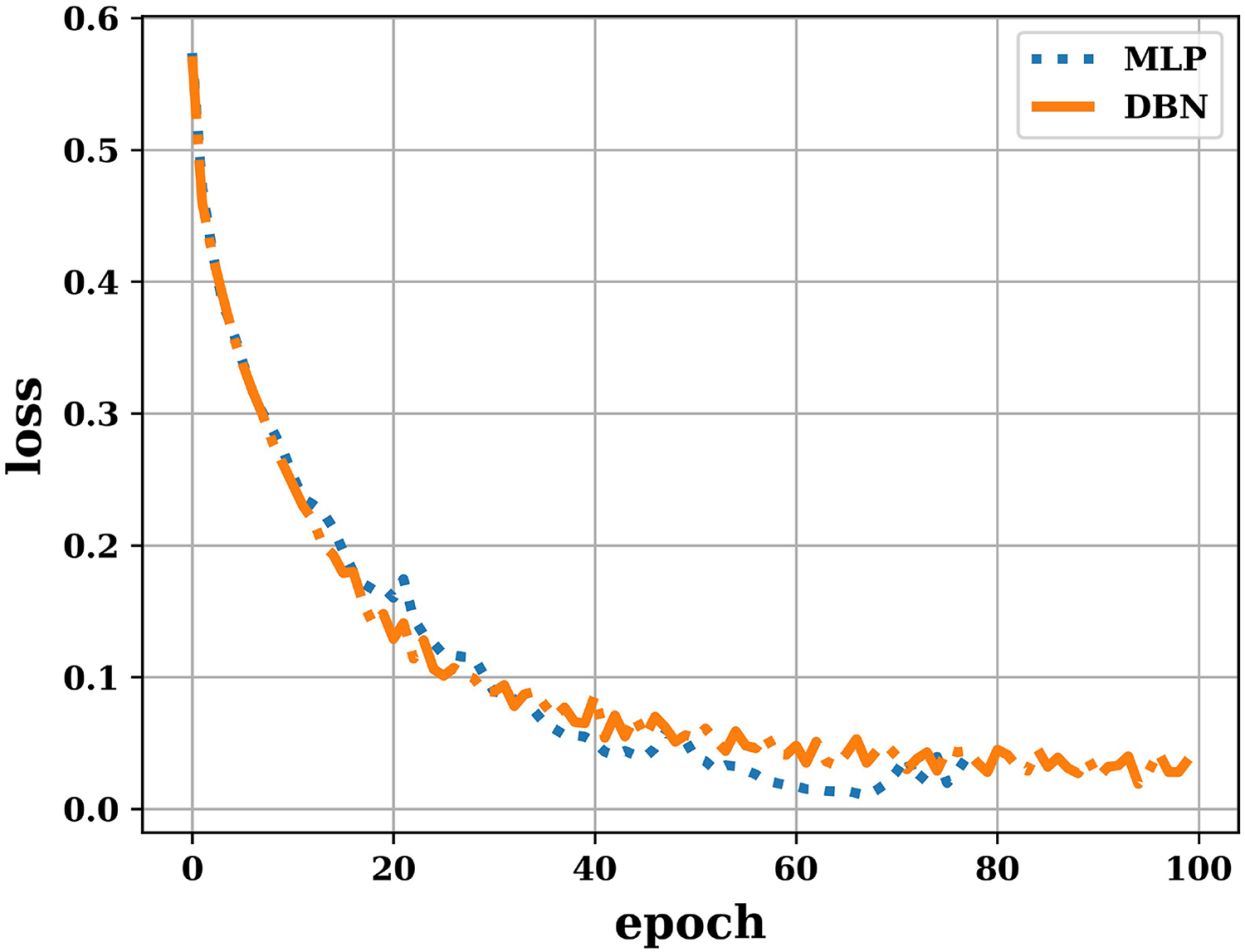
Training loss for deep learning models.

In the study, we have also used deep learning models (MLP and DBN) for allergen classification. The loss occurred during training of these models with varying epoch is shown in Fig. 3. The figure shows that as the number of epochs increase, the training loss for both the models decrease and attain a minimum values of 5% (approx.). This reduced value of training loss indicates the effective training of deep learning models.

The test results of various machine learning, deep learning and proposed model are presented in Table 3 in the form of True Negative (TN), False Positive (FP), False Negative (FN), True Positive (TP), Training Time and Testing Time (in seconds).

**Table 3:** Classification results of various learning models on the test dataset.

Model	TN	FP	FN	TP	Training time (sec.)	Testing time (sec.)
Logistic regression (LR)	553	158	148	598	0.5419	0.00204
k-NN	590	121	85	661	0.0027163	0.176971
Linear SVM (LS)	558	153	148	598	2.41266	0.23742
Polynomial SVM (PS)	609	102	176	570	1.2014	0.3266
SVM with RBF (RS)	588	123	109	637	0.81344	0.31443
Random forest (RF)	624	87	97	649	3.479488	0.07969
Adaboost (AB)	586	125	116	630	6.21499	0.089167
Gradient boosting (GB)	595	116	105	641	14.23084	0.004366
MLP	664	47	180	566	28.3818	0.0010564
DBN	622	100	84	651	48.268	0.00113
Extra tree (ET)	627	84	102	644	8	0.075486
XG boost (XB)	619	92	85	661	9	0.084128
Cat boost (CB)	622	89	83	663	56.2	0.094152
Proposed model (proposed)	634	77	81	665	56.416	0.12854

The lower values of False Positives and False Negatives for DBN, Extra Tree and CatBoost models, as shown in Table 3, resulted into the formation of proposed ensemble model, with an objective to further enhance the overall classification performance. The proposed model has reduced the number of FP and FN, as show in the table. The test results of each model are then used to compute the value of other performance parameters.

**Figure 4:**
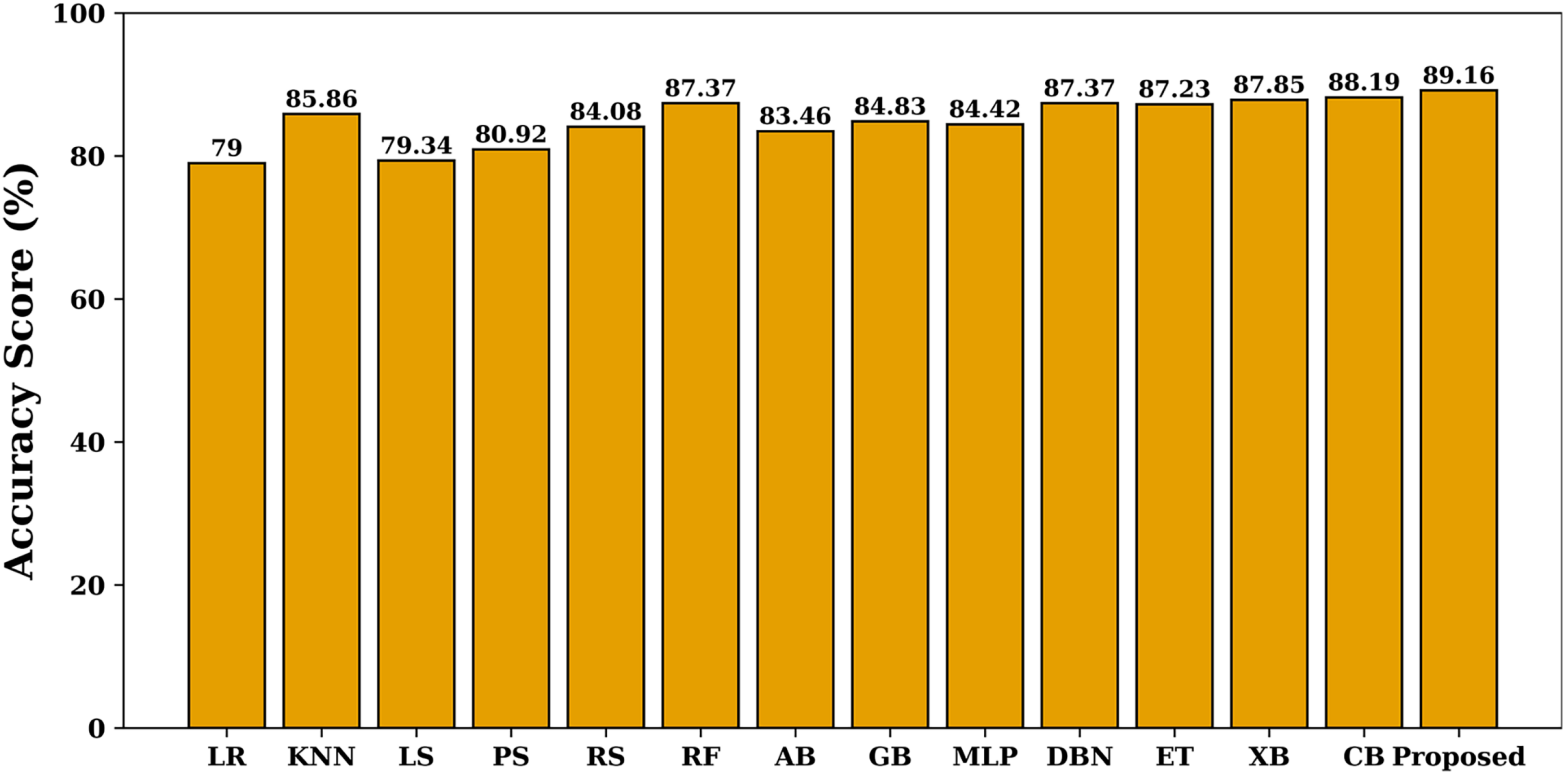
Allergen classification accuracy of various models.

**Figure 5:**
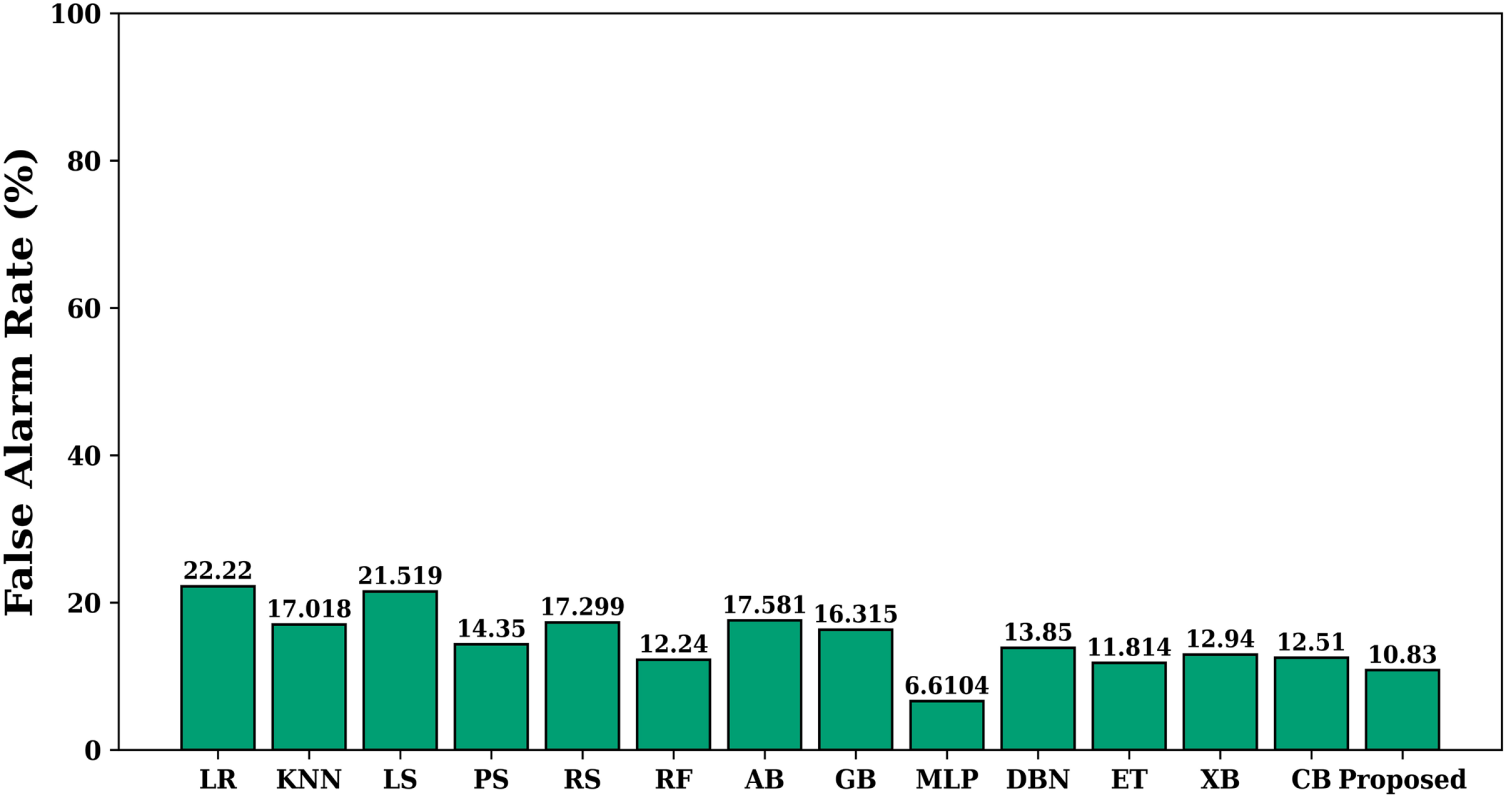
False alarm rate of various models.

The Fig. 4 shows the value of allergen classification accuracy of different models studied on the test dataset. Accuracy offers a gauge of how effectively the model can generate accurate assumptions about fresh and unseen data. A high accuracy rating means the model can generalize effectively and make precise assumptions about new data. The figure shows that the proposed model achieves maximum accuracy when compared with other models. The false alarm rate of each learning model is shown in Fig. 5. The figure shows that the proposed model has least false alarm rate of 10.83% than the other studied models (except MLP).

**Figure 6:**
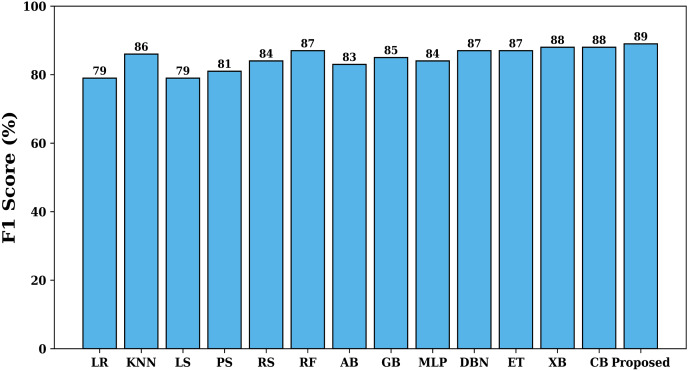
F1-score of various models.

The F1-score is a statistic that integrates recall and precision into one performance parameter for the model. It offers a fair assessment of the model’s capacity to recognize both positive and negative instances. When the cost of false positives and false negatives is not equal, this is especially crucial. The F1-score of various studied models is shown in Fig. 6. It is evident from the figure that the proposed model has an F1-score of 89%, which is maximum when compared with other studied models.

**Figure 7:**
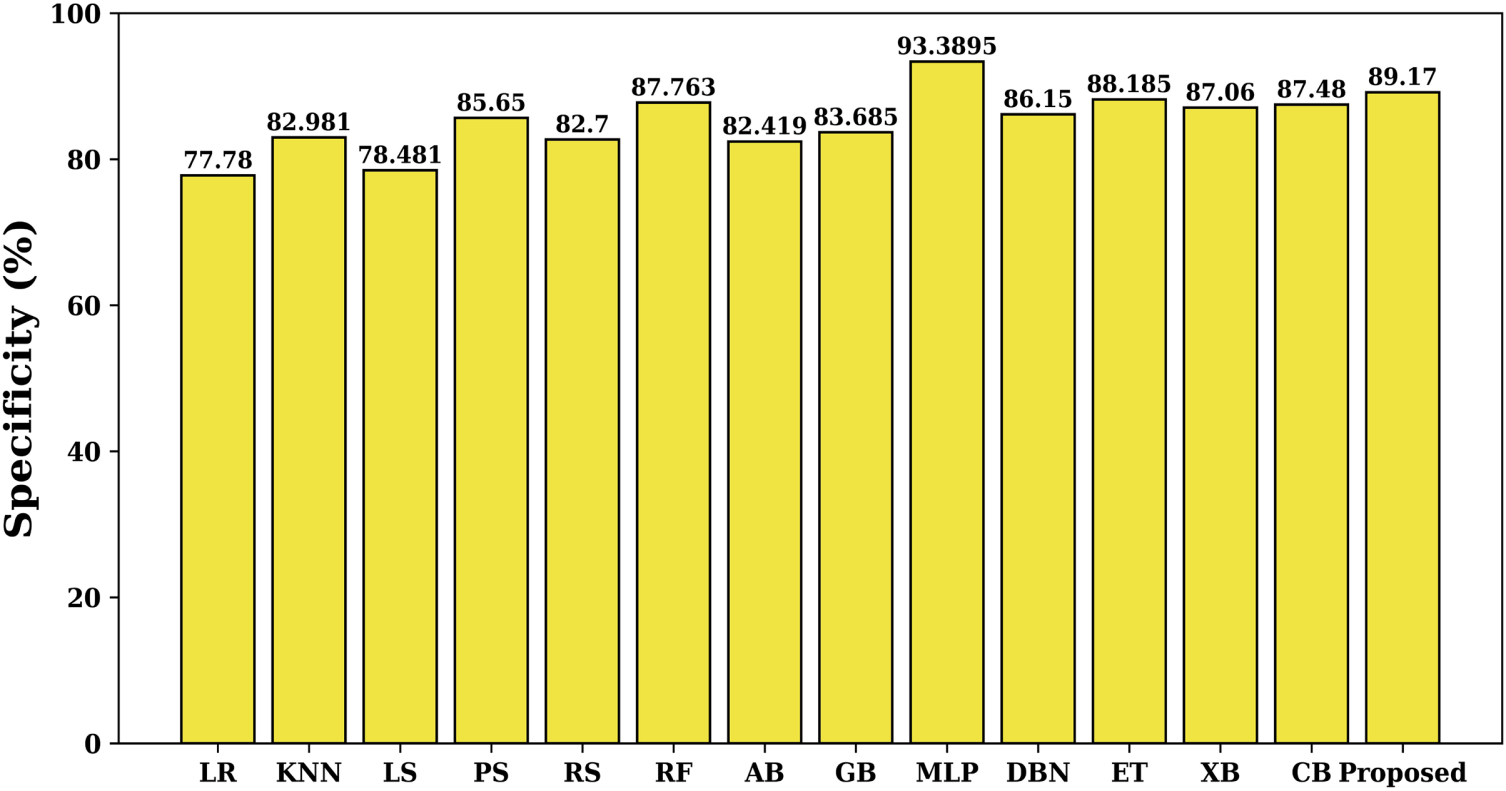
Specificity of various models.

Specificity quantifies the percentage of true negatives that the model properly detects, *i.e*., it tells us how well the model is able to detect undesirable circumstances. Figure 7 shows this specificity value for each studied model. Here again the proposed model has maximum specificity value of 89.17% than the other studied models (except MLP). The MLP model has maximum specificity value due to the lower value of false alarm rate. Besides MLP, the proposed model has maximum value of specificity than the other studied models.

**Figure 8:**
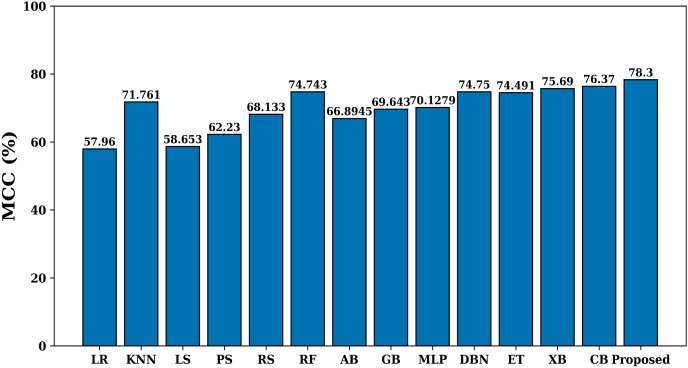
MCC of various models.

The significance of Matthews correlation coefficient (MCC) is that it does not depend on unbalanced datasets and gives a fair assessment of the model’s performance, accounting for both true positives and true negatives. The MCC runs from −1 to +1, with +1 denoting perfect agreement, 0 denoting predictions that are no better than random, and −1 denoting utter disagreement between the predicted and the actual values. The MCC value of each model on the test dataset is shown in Fig. 8. Here again the proposed model has maximum MCC value of 78.3%, which shows the effectiveness of proposed model for allergen classification.

**Figure 9:**
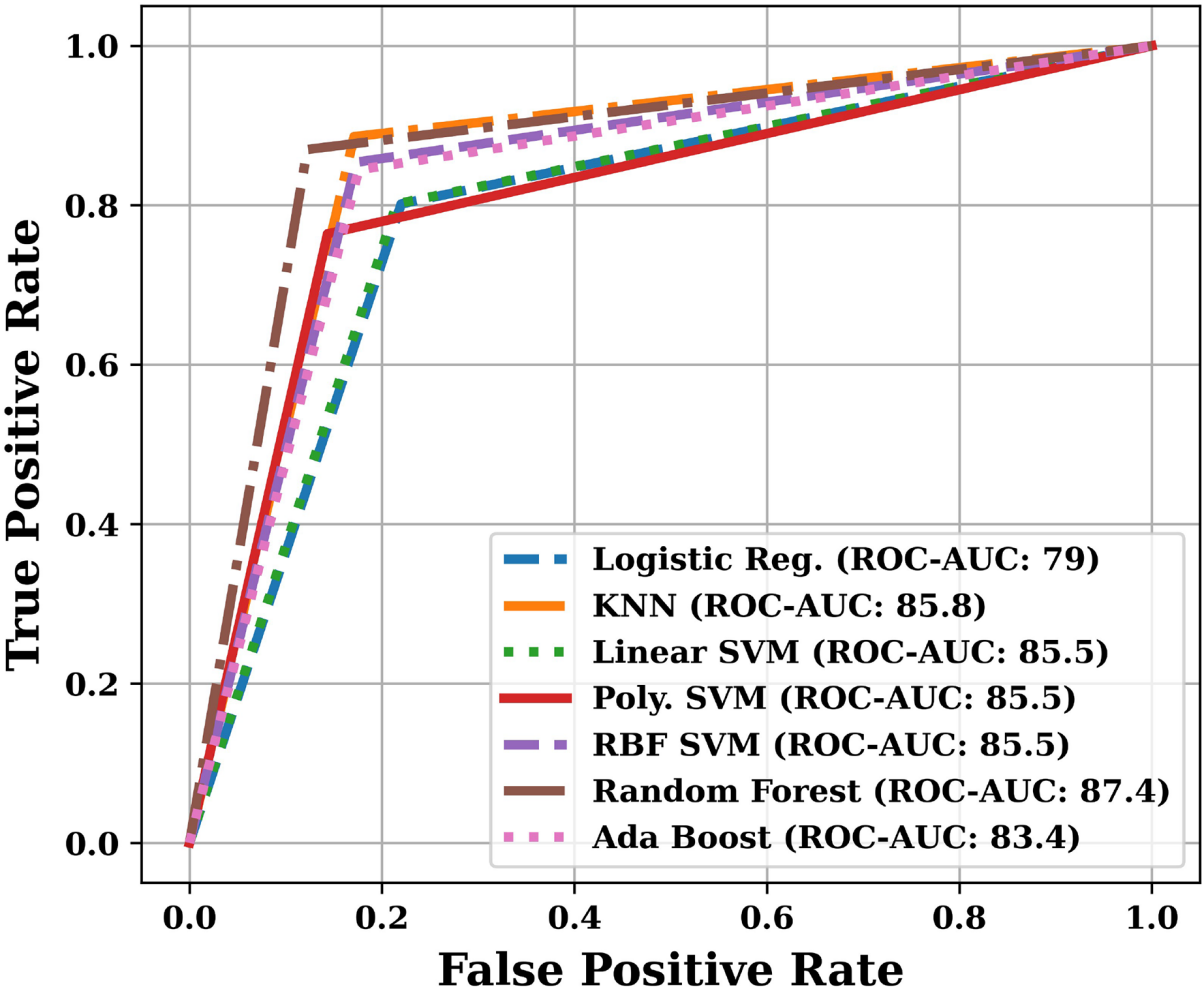
ROC curves of various models (Part-I).

**Figure 10:**
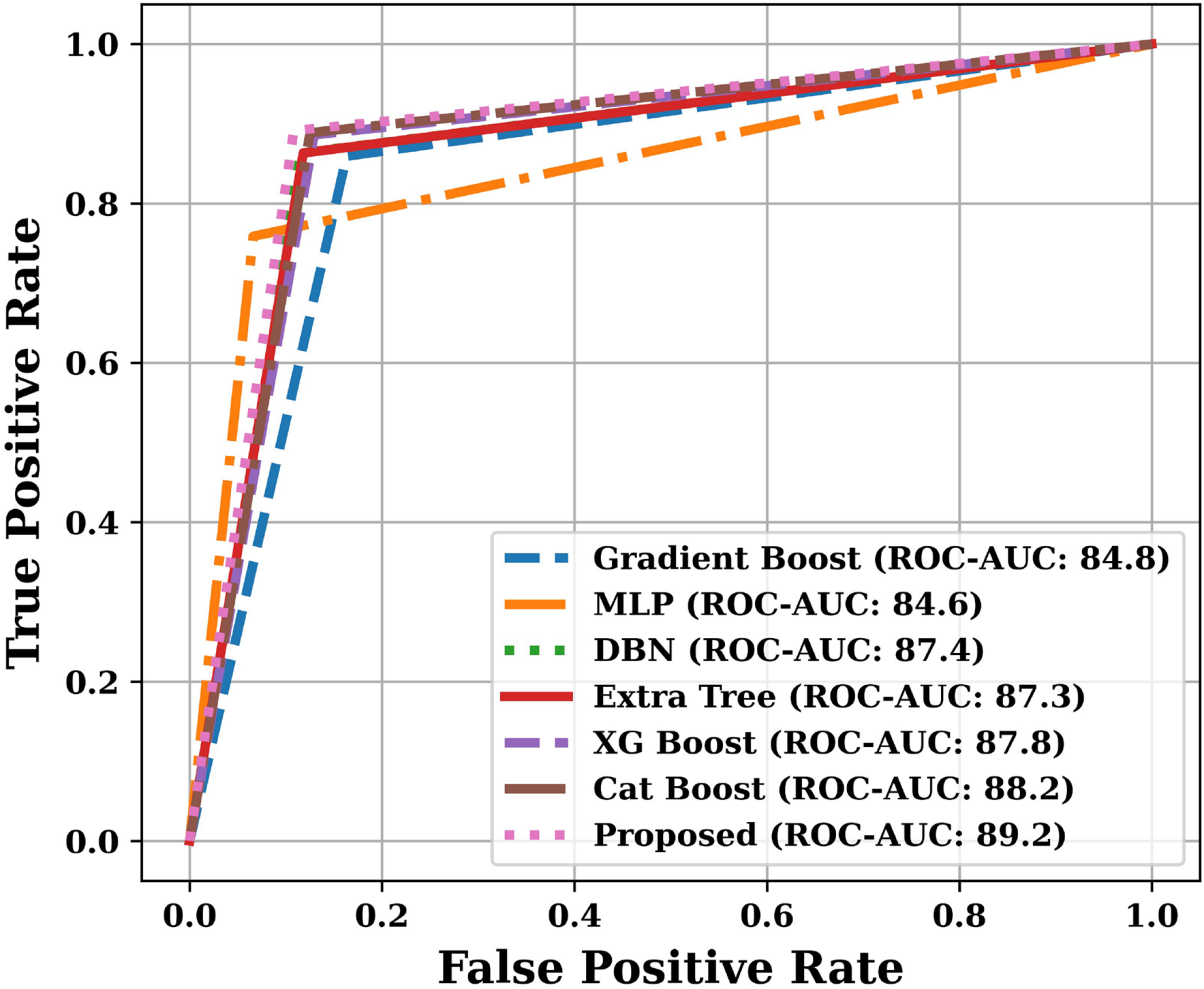
ROC curves of various models (Part-II).

**Figure 11:**
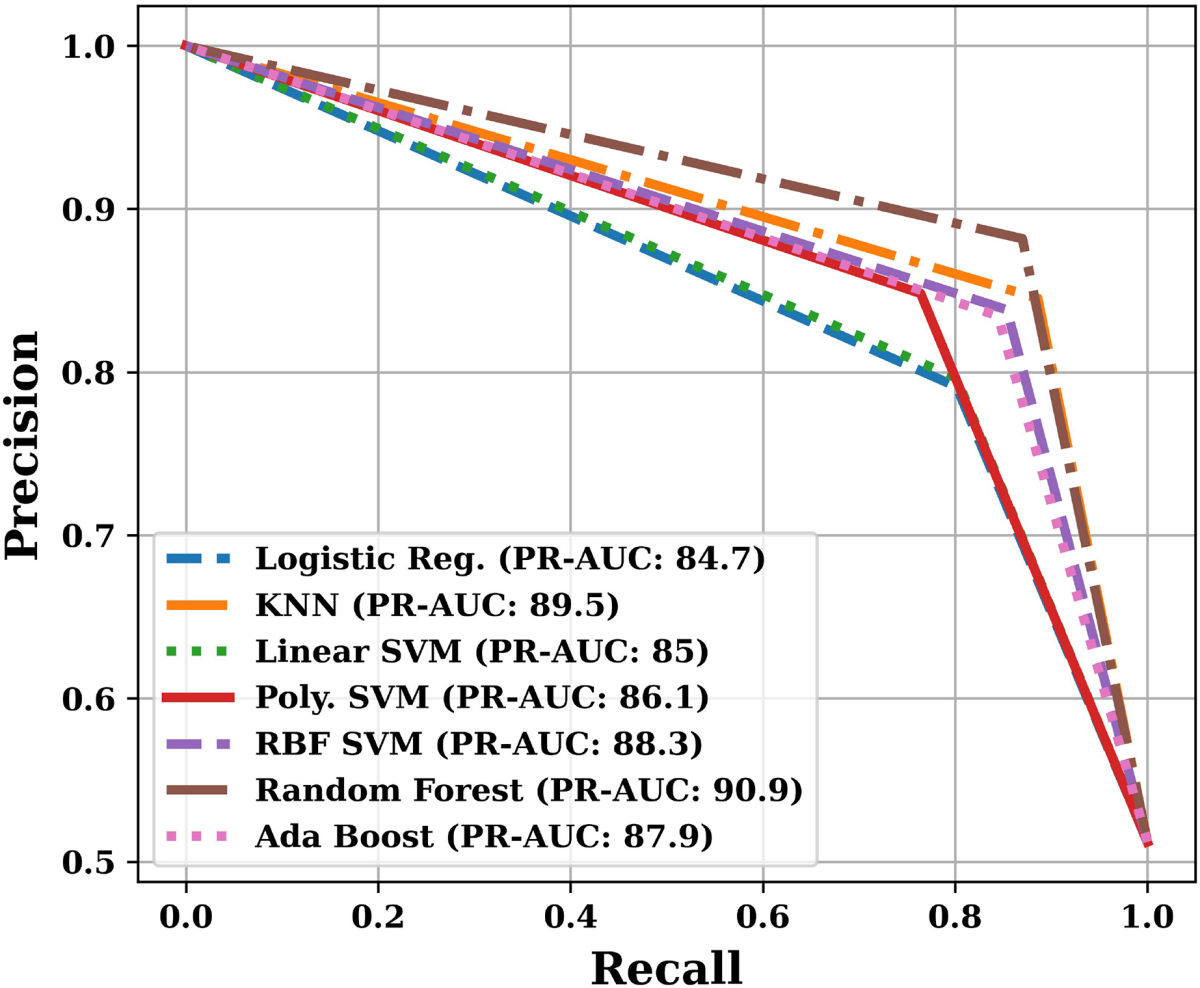
PR curves of various models (Part-I).

**Figure 12:**
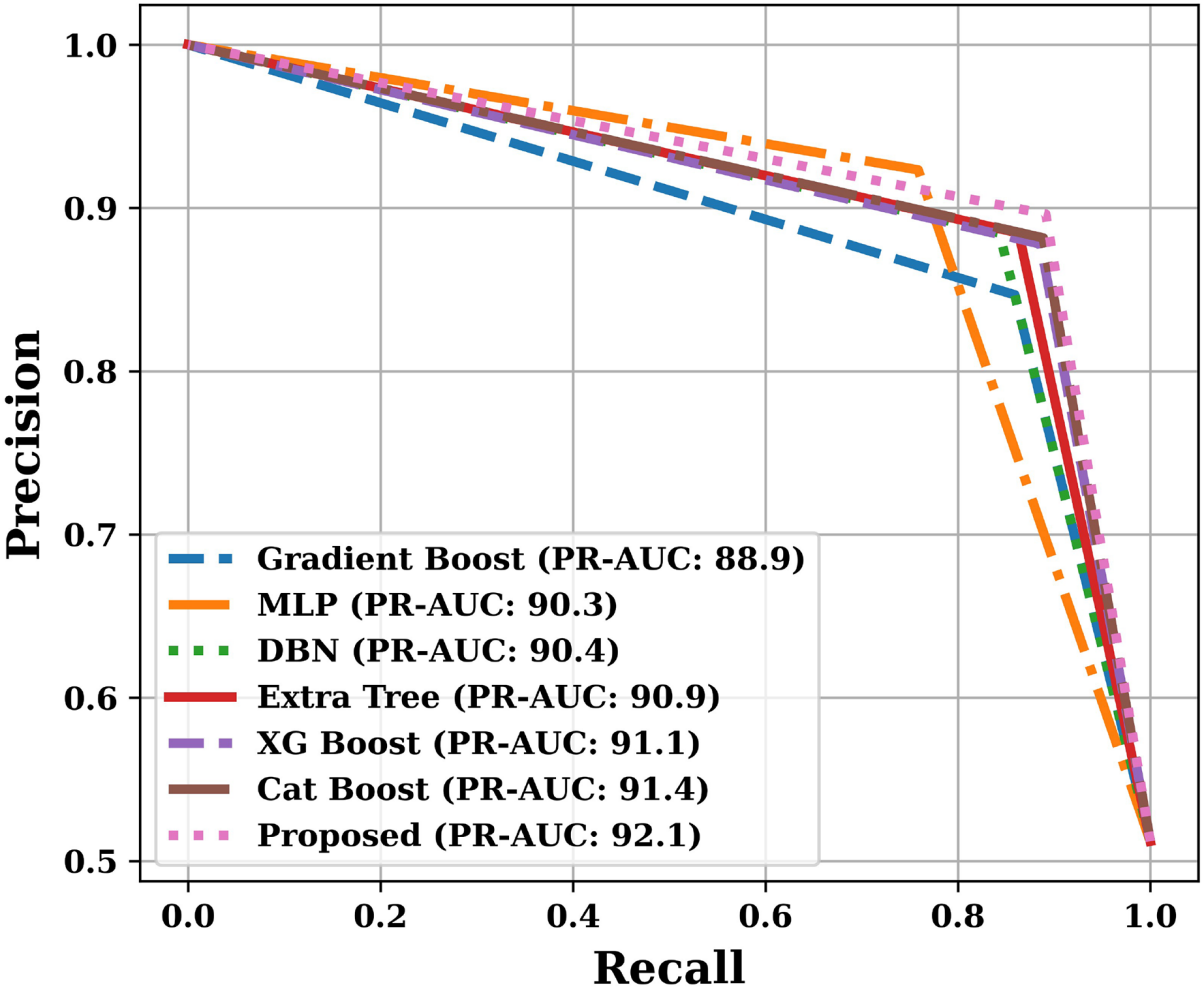
PR curves of various models (Part-II).

Apart from the already described performance parameters, we have also plotted ROC and PR curves for various models on the test dataset. Since the number of models are more, that is why we have presented the ROC and PR curves in two parts. The ROC curves are shown in Figs. 9 and 10, and the PR curves are shown in Figs. 11 and 12. We have also calculated the Area Under each Curve (AUC) and is mentioned in each figure. Closer the value of AUC to 100%, more efficient is the classification model. After analysing the figures, it is clear that the proposed model has an area under the ROC curve value of 89.2% and area under the PR curve value of 92.1%, which is maximum when compared with the other studied models.

The combined analysis of all the presented results clearly reveals that the proposed model effectively detects allergen and non-allergen protein sequences. The proposed model has maximum value of detection accuracy, F1-score, MCC and area under the ROC and PR curves.

### Performance comparison

**Figure 13:**
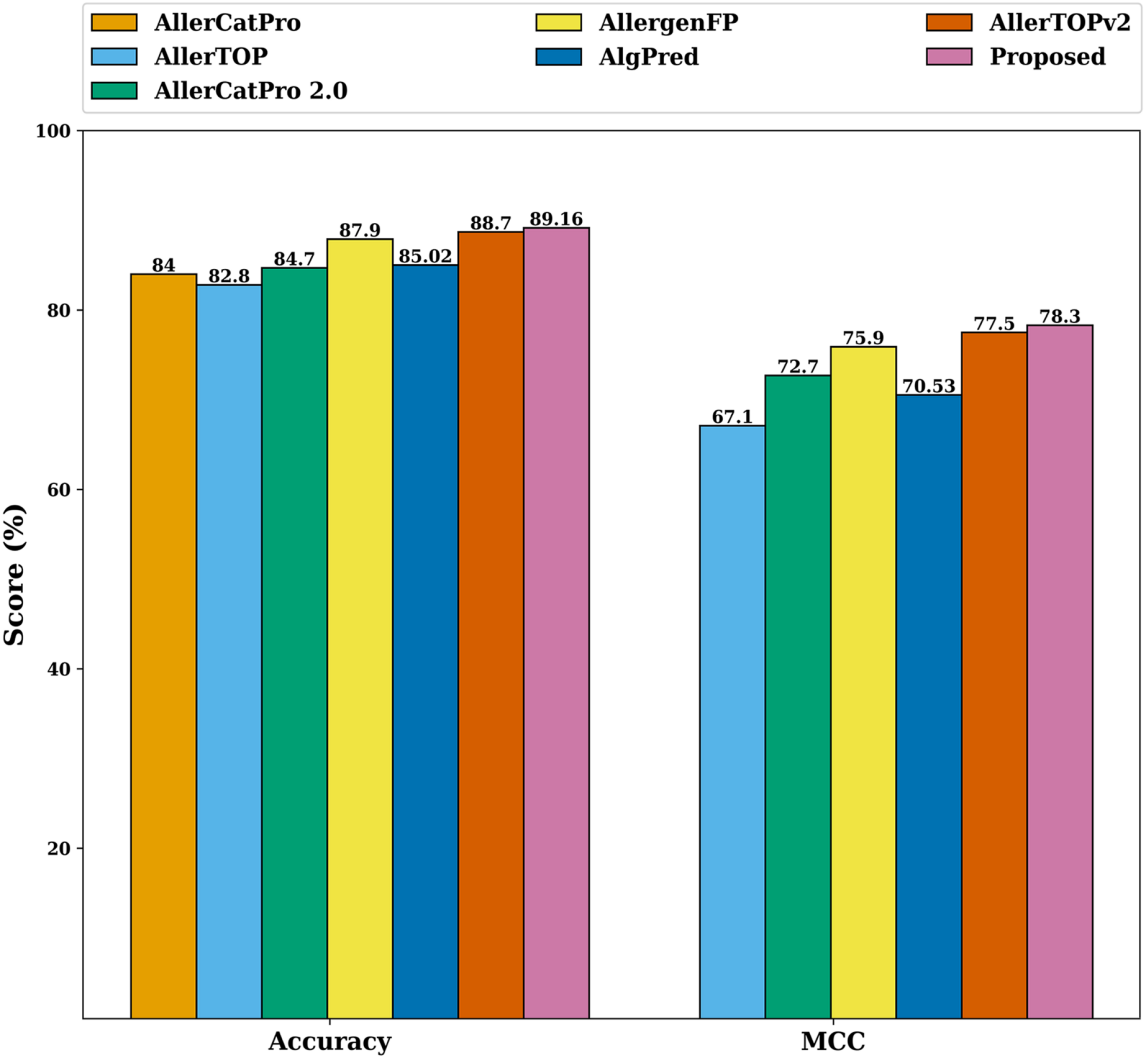
Comparison of the proposed model with related work.

To further specify the significance of the proposed model, we have compared its performance with AllerTOP (Dimitrov, Flower & Doytchinova, 2013), AlgPred (Saha & Raghava, 2006), AllergenFP (Dimitrov et al., 2014b), AllerTOPv2 (Dimitrov et al., 2014a), AllerCatPro (Maurer-Stroh et al., 2019) and AllerCatPro 2.0 (Nguyen et al., 2022). We have evaluated the performance of the proposed model on different datasets (independent from the one described in section 2.4) used in AllerTOP, AlgPred, AllergenFP, AllerTOPv2, AllerCatPro, and AllerCatPro 2.0 respectively. The performance comparison is shown in Fig. 13, where it is clear that the proposed model outperforms the specified state-of-the-art literature techniques for allergen classification. Figure 13 shows that the proposed model has an average allergen detection accuracy of 89.16% and MCC of 78.3%, which is highest when compared with the other state-of-the-art literature techniques. The comparative study also shows that the model is free from overfitting issues and is stable.

## Conclusion

This article presented the analysis of various machine learning and deep learning techniques to propose an ensemble learning based approach for the identification of protein allergens. The article also highlighted various entities and concepts related to allergy control. In this article, we also described various protein sequence properties like physicochemical, molecular, antimicrobial and other characteristics to extract the feature vectors, giving rise to the dataset used for training and testing of intelligent techniques. The performance evaluation of various machine learning, deep learning techniques and proposed mechanism revealed that the proposed mechanism outperforms the other discussed techniques and achieved an allergen detection accuracy of 89.16%. The performance of proposed mechanism was also compared with the state-of-the-art literature techniques, which used the similar dataset as used in this study, and the results prove that the proposed mechanism outperforms the other techniques.

Even though the proposed model outperforms the other related techniques, the overall accuracy can be further enhanced. So, in the future, various approaches like increasing the size of dataset, use of different optimization and regularization techniques, *etc*., can be used which will further enhance the performance of the proposed mechanism. Furthermore, the diversity of the dataset can be increased in the future by adding data from different geographical regions for increasing the precision of the proposed model.

## Supplemental Information

10.7717/peerj-cs.1622/supp-1 Supplemental Information 1 Allergen Non-Allergen Data. First column represents protein sequences followed by features associated to that sequence. The last column 'allergen' represents the target. 1 represent allergen, 0 represent non-allergen.

10.7717/peerj-cs.1622/supp-2 Supplemental Information 2 Implementation Code.

## Additional Information and Declarations

### Data Availability

The following information was supplied regarding data availability: The raw data and code are available in the Supplemental Files.
